# Trauma-Informed Care in the health-care for transgender and gender-diverse adults: a scoping review

**DOI:** 10.3389/fpsyt.2025.1577225

**Published:** 2025-06-19

**Authors:** Hannah Borcherding, Marlena L. Itz, Bernhard Strauß, Timo O. Nieder

**Affiliations:** ^1^ Institute for Sex Research, Sexual Medicine, and Forensic Psychiatry, University Medical Center Hamburg-Eppendorf, Hamburg, Germany; ^2^ Institute of Psychosocial Medicine, Psychotherapy and Psychooncology, University Hospital Jena, Jena, Germany

**Keywords:** Trauma-Informed Care (TIC), trauma-informed, transgender and gender-diverse (TGD), trans, gender incongruence, healthcare

## Abstract

Transgender and gender-diverse (TGD) individuals frequently experience discrimination, minority stress, microaggressions, and traumatic events, including physical and sexual violence. It is essential to consider these traumatic experiences in clinical practice, as negative experiences within the health-care system can deter TGD individuals from seeking necessary support. However, many health-care professionals lack adequate training to respond effectively to the needs of TGD individuals. Trauma-Informed Care (TIC) presents a potential solution by encouraging professionals to recognize trauma, prioritize safety and transparency, and adapt their behavior to minimize distress. This scoping review aims to provide an overview of the existing empirical literature for the application of TIC in TGD health-care settings. A comprehensive literature search was conducted using the EBSCO, PubMed, and Scopus databases to identify studies examining TIC specifically within TGD health-care contexts. The review followed the PRISMA checklist extension for scoping reviews. Inclusion criteria required that studies analyze original data on the implementation of TIC in TGD health-care settings. The included studies were analyzed to assess the TIC principles included and their impact on health outcomes. The review identified four studies that met the eligibility criteria. Findings suggest that while TIC can provide significant benefits in fostering safe and affirming health-care environments for TGD individuals, the literature remains mixed and scarce. Methodological diversity and varying definitions of TIC complicate the synthesis of results. Gaps in research and inconsistencies in its application were highlighted. The findings underscore the potential of integrating TIC principles into health-care for TGD populations, as current frameworks often overlook their unique needs. For future research it seems crucial to conduct effectiveness studies of TIC such as randomized-controlled trails, standardize TIC definitions, develop robust outcome measures and explore TIC in various contexts. This review highlights the potential of TIC in the healthcare for TGD individuals while emphasizing the need and providing directions for further research.

## 1 Introduction

According to current figures, around 0.5% to 1.3% of the population experiences gender incongruence, i.e. the mismatch between a person’s sex assigned at birth and their gender, as seen in transgender and gender diverse people (TGD) (1, 2). This can lead to gender dysphoria, which is defined as significant discomfort or distress due to this mismatch and is classified as a disorder in the DSM-5-TR (3). Formerly a rare diagnosis, in the last decades, there has been a sharp rise in prevalence (4, 5), a development that has presented both healthcare and research in this area with major challenges. Research has increased dramatically in recent years leading to an array of new developments and insights (6). However, there are still major gaps and controversies both in relation to research as well as the care of TGD people.

TGD people are a minority. Therefore, this group is more likely to experience trauma, and violence (7, 8), and to have negative experiences within the health-care system (9, 10). As a result TGD people are significantly more likely than cisgender people to encounter traumatic events in their lives and to develop posttraumatic stress disorder (PTSD) (8). A traumatic event according to the DSM-5-TR is defined as a life-threatening event, serious injury or sexual violence that threatens a person’s sense of physical or psychological safety (3). Adding to this, according to Minority Stress Theory (11, 12), members of sexual and gender minority groups are often disproportionately affected by marginalization, stigmatization, and discrimination. Examples of this are devaluation, micro-aggression, verbal abuse, sexual and physical victimization, invalidation, transphobia, and structural oppression (13, 14). In frameworks like minority stress theory, trauma is often conceptualized in a broader sense than in the DSM-5-TR. Here trauma extends beyond isolated, catastrophic events to include ongoing, cumulative experiences – such as persistent discrimination, social exclusion, or microaggressions – that might not meet the DSM-5-TR criteria but can still profoundly diminish a person’s sense of safety and well-being. This expanded view considers a traumatic experience not only as the event itself but also as the subjective impact it has on the individual (11, 15). It follows, that these experiences of minority stress can also be traumatizing, even if those who endure them do not necessarily meet the diagnostic criteria for PTSD (16). Aside from PTSD, traumatic events and prolonged stress linked to LGBTQ+ based discrimination were found to be associated with conditions such as attachment anxiety and avoidance, emotion dysregulation and dissociative symptoms (17), as well as to a range of other psychological and physiological challenges such as vigilance, rumination, and alterations in stress hormone levels (18). Therefore, it is critical for health-care professionals (HCPs) to understand the impact of gender minority status on this group, as approaches that consider their specific experiences hold significant potential for improving care. This highlights the importance of taking into account trauma and discrimination experienced by this group when providing healthcare (19).

One approach which aims to achieve this, as well as to enhance the health-care system’s inclusivity and accessibility for TGD individuals, is Trauma-Informed Care (TIC) (15). TIC does not offer specific trauma-related treatments or interventions. Instead, it encourages a perspective that views clients through the lens of trauma and their lived experiences, such as minority stress, understanding and recognizing trauma and adapting accordingly. TIC has been proposed as a possible complement to existing evidence-based interventions as well as to be incorporated into organizational structures with the aim of providing a more holistic understanding of clients’ problems, strengths, and needs by taking into account their experiences (20–22). A trauma-informed approach can be applied at all levels of an organization or system, from the person who opens the door, to the practitioner, as well as structures and policies (15). In addition, TIC can help to reduce stigma and pathologization surrounding trauma-related symptoms and behaviors (23), which is particularly relevant for TGD individuals who have a history of discrimination and pathologization within the health-care system (7).

**Figure 1:**
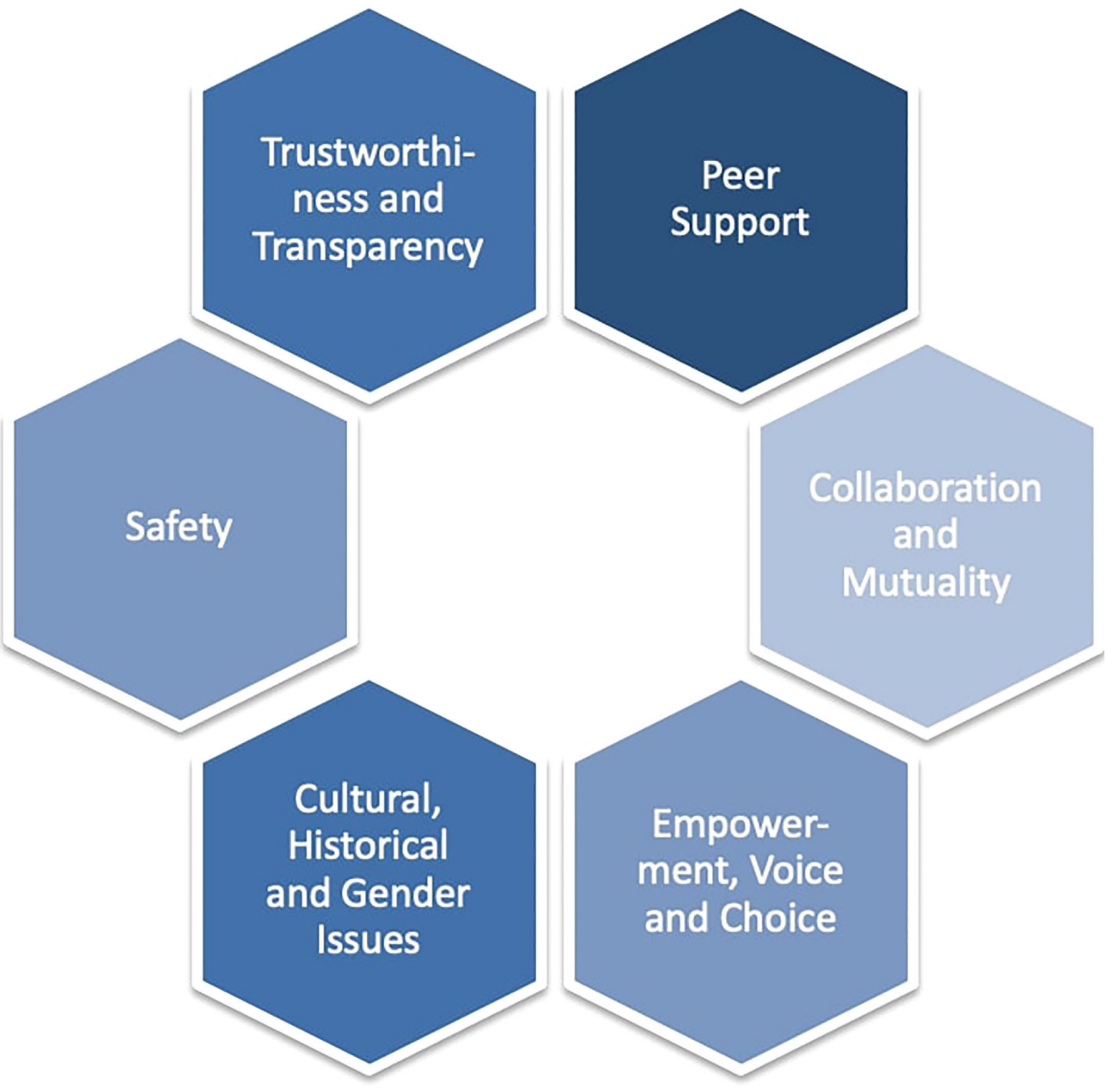
Trauma-informed care principles.

Since substance disorders are also commonly paired with traumatic experiences, the Substance Abuse and Mental Health Service Administration (SAMHSA) has developed a concept of a trauma-informed approach that is widely used as a foundation for research and practice in the field of TIC (15). The key principles vary slightly across literature but most authors, like SAMHSA (15), have settled on six principles (see **Figure 1** ‘Trauma-Informed Care Principles’). These principles are *safety* (*physical* and *emotional/psychological*), *trustworthiness and transparency* (i.e. with regard to making decisions, building relationships with clients), *peer support*, *collaboration and mutuality* (focusing on collaboration and equalizing the power imbalance), *empowerment, voice and choice* (building on individuals’ resources and fostering resilience and self-determination), and lastly, *cultural, historical, and gender issues* (including a perspective that considers intersectionality and historical trauma). The principles outlined explicitly apply not only to therapeutic interactions but span across all individuals involved in care, as well as the organization as a whole (e. g. in leadership, policies, cross sector collaboration). In a theoretical paper, Levenson et al. (24) transferred the concept of TIC to the context of LGBTQ+ people, which can provide insight into the potential applications of TIC in the care for this group. With regard to the principle of *safety*, the paper emphasizes that HCPs should for example ask about preferred pronouns and be cautious about making assumptions about clients’ sexual and gender identity. Furthermore, in the context of *trustworthiness and transparency*, HCPs should avoid ambiguity and vagueness and show a non-judgmental, destigmatizing, depathologizing and consistent attitude. In addition, HCPs should help clients to find and use *peer support* resources (online or in person) such as support groups, websites, or social media platforms. According to Levenson et al. (24), *collaboration and mutuality* can be promoted in contact with LGBTQ+ clients, for example by requesting feedback from clients, setting individualized shared goals, and emphasizing that the HCP and client work together as a team. Regarding *empowerment, voice and choice*, HCPs should adapt to the client’s pace of self-opening and support their autonomy and independent decision-making. In the context of *cultural, historical and gender issues*, it is emphasized that historical traumas of marginalized groups, the socio-political context, and intersectionality should be considered (24).

The use of TIC has been found to be beneficial in many different contexts such as gynecologic oncology (25), emergency medicine (26), and pediatric obesity management (27). A systematic review supports its use for psychological outcomes (28) and another its implementation on an organizational level, e. g. regarding leadership (29). Based on these promising results associated with TIC in various contexts and in light of the aforementioned substantial prevalence of traumatic experiences in TGD individuals and the consequent effects on their psychological and physiological well-being (17), many authors advocate for the utilization of TIC in the care of TGD individuals, for example, in reproductive health-care settings (30), pediatrics (31), imaging care (32), and mental healthcare (33). While relevant (inter)national guidelines on this topic tend to be vague and do not explicitly recommend TIC, they do imply a trauma-informed approach in some of their recommendations (1, 34–36). Furthermore, Levenson et al. (24) demonstrated that, in theory, the concept of TIC can be transferred to the context of care for TGD people. In addition, TIC has been found to be used in some programs for LGBTQ people in the USA already (37). However, even though there seems to be a strong alignment between TIC principles and the needs of TGD individuals, few studies have examined its application in this context. Moreover, no comprehensive reviews exist that investigate the existing empirical literature on the application of TIC in TGD care. Consequently, this scoping review aims to compile and analyze the existing literature on this topic. Specific emphasis will be placed on the individual TIC principles upon which each of the studies have based their respective analyses.

## 2 Methods

This review was conducted in accordance with the PRISMA checklist extension for scoping reviews (38) and methodological instructions for scoping reviews (39). The theoretical framework and methodological planning were carried out by the first and last author of the paper. The literature review was conducted by the first author.

### 2.1 Search strategy

The research was conducted in three online research tools EBSCO, PubMed, and Scopus in April 2024. Search terms regarding gender incongruence were combined with search terms relating to TIC. The following search terms were used, searching by subject terms or title and abstract: (trauma informed) AND (transgender OR gender incongruence). In EBSCO the databases APA PsyInfo, APA PsycArticles, MEDLINE complete, CINAHL complete, Psychology and Behavioral Sciences Collection and SocINDEX were included in the search and the filter “only Academic Journals” was applied. In Scopus the filters regarding the type of publication “Article” and “Review” were used. To obtain a comprehensive overview of the existing literature, no filter regarding the publication date were applied during the search.

### 2.2 Eligibility criteria

The main inclusion criterion was that studies needed to examine the application of TIC specifically in the care of TGD individuals. Additionally, texts were excluded if they did not analyze original data to investigate the implementation of TIC in the healthcare of TGD individuals.

### 2.3 Selection process and data extraction

**Figure 2:**
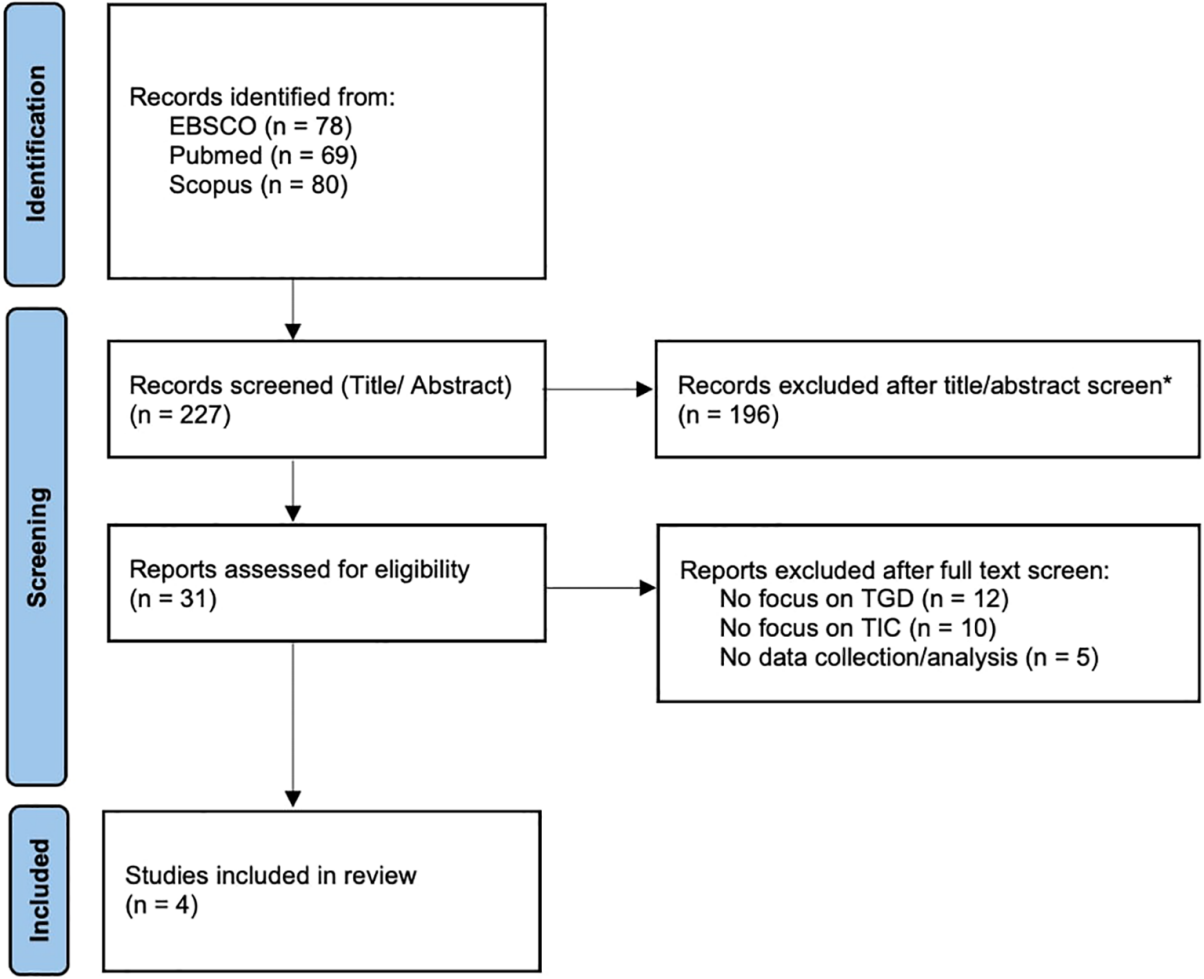
PRISMA Flow Diagram. *Excluded articles due to irrelevance to the topics of the scoping review. Source: Adapted from Page et al. (40).

The searches conducted across PubMed, Scopus, and EBSCO resulted in a total of 227 records (for details see **Figure 2** ‘PRISMA Flow Diagram’). The titles and abstracts of the studies found were screened for relevance according to the eligibility criteria by the first author. Thereupon a thorough full-text screening was performed by the first author on 31 articles to assess their eligibility for inclusion, resulting in four remaining for analysis. As this is a scoping review, no studies were excluded due to their quality or risk of bias (39). Possible biases however should be considered when interpreting the results and will be analyzed in detail in the discussion. The full texts of the included studies were analyzed, with particular attention given to the assessment and definition of TIC and the study results regarding the application of TIC in the healthcare for TGD. In the analysis, the principles outlined by SAMHSA were employed to examine the components of TIC utilized across the various studies. The derived information was gathered in **Table 1** (‘Results’).

**Table 1:** Results (*validated; ^+^subscale(s) of a validated questionnaire; °not validated; ^1^Trauma-Informed Care; ^2^transgender and gender-diverse; ^3^interpersonal violence survivors; ^4^chronic physical health problems, ^5^health-care professional).

Study & Year	Sample	Design & Analysis	Assessment of Outcomes	TIC^1^ Components & Assessment	Study Results Related to TIC^1^
Antebi-Gruszka & Scheer (41) 2021	298 LGBTQ (41.6% TGD^2^) IPVS^3^ (online groups)	Online survey; regression models (IV: TIC^1^ components; DV: outcomes)	- Health (PTSD*, depression*, substance use°, somatic symptoms^+^, sexual risk°, CPHP°^4^) - Psychosocial (shame^+^, loneliness*, cognitive reappraisal^+^, empowerment*)	- Agency; information; connection; strengths; cultural responsiveness & inclusivity (TIP^1^ scales^+^) - Minority stress (minority stress sensitive TIC^1^ scale^)	- Agency negatively related to PTSD depression, shame & loneliness - Connection positively related to sexual risk - Cultural responsiveness & inclusivity positively related to depression, PTSD & CPHP^4^
Hall & DeLaney (42) 2021	100 TGD^2^ (online support groups)	Online survey; thematic analysis (inductive)	Open questions about their communities & experiences in health-care system	Trust & safety; environmental & physical safety; choice & collaboration; empowerment; cultural & gender issues	- TIC^1^ provides a framework for TGD^2^ adults needs within mental health services & community support
Lacombe-Duncan et al. (43) 2021	26 trans women & 10 HCPs^5^ (networks & emails)	Focus groups & interviews; thematic analysis (in-/deductive)	Semi-structured focus groups with trans women & semi-structured interviews with HCPs^5^	TIC^1^ as a whole: affirmation & tolerance, no stigma/judgement, deconstructing power dynamics, contextualizing issues	- Trans-positive TIC^1^ found to be one of three facilitators to access HIV healthcare for trans women (TIC^1^ most prominent facilitator)
Scheer & Poteat (44) 2018	239 LGBTQ (42.7% TGD^2^) IPVS^3^ (online groups)	Online survey; structural equation modeling (TIC^1^ links to health via mediators)	- Mediator (shame^+^, emotion regulation^+^, empowerment*, social withdrawal*) - Mental health (depression*, PTSD*) - Physical health (CPHP°^4^, somatization^+^)	TIC^1^ as a whole: agency; information; connection; strengths; minority stress (TIC^1^ scales^+^; minority stress sensitive TIC^1^ scale^)	- TIC^1^ linked to greater empowerment, emotion regulation & lower social withdrawal, but not shame - Hypothesized indirect associations (mostly) insignificant

## 3 Results

From the four studies included in the current review the two studies by Antebi-Gruszka and Scheer (41) and Scheer and Poteat (44) use the same data. Because of the different research questions and analyses however both studies were included.

### 3.1 Study characteristics

Antebi-Gruszka and Scheer (41) examined the association of the perception of receiving TIC with health and psychological well-being in a sample of LGBTQ people who had survived intimate partner violence (IPV) and sought trauma-related services (e. g. therapy, legal counselling) in an online survey. Hall and DeLaney (42) aimed to identify trauma-sensitive strategies to enhance the care for TGD adults. In an online survey they asked TGD individuals open questions (“Think about the Communities to which you belong. What Qualities would make these (…) more supportive of you in the Future?”; “Think about the best Experience you Have Had During a Mental Health Appointment. What Made this Experience positive for you?”). Lacombe-Duncan et al. (43) used semi-structured focus groups with trans women on experiences accessing HIV healthcare and desired HCPs characteristics and semi-structured interviews with HCPs on barriers and facilitators to providing HIV prevention and other services to trans women. Scheer and Poteat (44) used a hypothesized mediation model to examine how LGBTQ IPV survivors’ perception of receiving TIC was associated with mediators and how those were associated with mental and physical health outcomes.

The studies analyzed differ greatly in terms of the research question, analysis, and outcomes. While Antebi-Gruszka and Scheer (41) and Scheer and Poteat (44) used questionnaires to collect data on health and psychosocial outcomes, Hall and DeLaney (42) asked two open questions, and Lacombe-Duncan et al. (43) conducted semi-structured focus groups and individual interviews. Furthermore, in contrast to the other studies, Lacombe-Duncan et al. (43) also surveyed HCPs in addition to those affected. Moreover, whereas Hall and DeLaney (42) and Lacombe-Duncan et al. (43) collected qualitative data, the other two studies gathered quantitative data. It should also be noted that all included studies were conducted in North America. While Lacombe-Duncan et al. (43) collected data in Canada and Hall and DeLaney (42) in New York, the precise geographic origins of participants in the two online studies are less clear. However, based the location of the research teams and data collection, it is reasonable to assume that the samples in these studies were also predominantly North American. This assumption is further supported by the fact that participants in all four studies were predominantly White. The socioeconomic background was reported only in Antebi-Gruszka and Scheer (41) and Scheer and Poteat (44), where participants appear to represent a predominantly middle-class demographic (with over 40% agreeing to the statement “I can pay my regular bills, but a bill that was bigger than usual would cause hardship.”). These factors should be considered when interpreting the results of the synthesis and analysis, which can be found in **Table 1** (‘Results’).

### 3.2 Trauma-Informed Care in the analyzed studies

As previously described, the definition provided by SAMHSA (15) is widely used within the context of TIC, even though there are numerous definitions and frameworks for this concept (45). For the purpose of enhancing comparability and interpretation, the following analysis will examine the varying definitions of TIC in the studies analyzed, based on the conceptual framework of SAMHSA’s TIC principles (15).

#### 3.2.1 Assessment of Trauma-Informed Care

The Trauma-Informed Practice (TIP) Scales (46) used by Antebi-Gruszka and Scheer (41) and Scheer and Poteat (44) are based on exploratory factor analysis from a survey, expert interviews, and focus groups (47). Not all TIC components (organized as subscales) of the TIP Scales (46) can be clearly assigned to a SAMHSA principle (15). Notably, Antebi-Gruszka and Scheer (41) did not include the subscale “Parenting” and Scheer and Poteat (44) did not include “Cultural Responsiveness and Inclusivity” and “Parenting” in their respective analyses. Additionally, these two studies included information on minority stress-related TIC in their analysis through a corresponding questionnaire (e.g., “Staff/individual providers ask about LGBTQ-specific forms of discrimination that I have experienced”; “Staff/individual providers are knowledgeable about LGBTQ issues”; these questions were not included in the original paper and were provided to the first author by Dr. J. R. Scheer). In contrast to Antebi-Gruszka and Scheer (41), who analyzed the individual components of the construct TIC in relation to the outcomes, Scheer and Poteat (44) looked at TIC in general based on the questionnaires. During the thematic analysis, Hall and DeLaney (42) found that their qualitative data fit well into the TIC framework, so this concept was used as a framework for their analysis. Lacombe-Duncan et al. (43) were also able to identify elements of TIC in the thematic analysis and highlighted individual aspects of the framework that were found in the qualitative data.

#### 3.2.2 Mapping the Trauma-Informed Care principles within the SAMHSA framework

In the TIP Scales (46), upon which Antebi-Gruszka and Scheer (41) and Scheer and Poteat (44) base their findings, the items within the subscale “Agency” can be associated with different TIC principles outlined by SAMHSA (15). One of these principles is *emotional safety*, which is reflected in items such as “Staff respect my privacy.” (47). Notably, *physical safety* is not included in the TIP Scales. Hall and DeLaney (42) differentiate the SAMHSA (15) principle of s*afety* into two categories: “emotional trust and safety” and “environmental and physical safety.” In the context of *emotional safety*, it is highlighted that participants should feel secure in expressing themselves, that they should be accepted and validated, and that no assumptions should be made regarding their gender identity by HCPs. In the context of *physical safety*, topics such as the accessibility of restrooms are discussed. Moreover, Lacombe-Duncan et al. (43) stress the need for HCPs to be affirmative towards gender identities and maintain tolerance, even in the face of aggressive behaviors.

Several items from the subscale “Agency” of the TIP Scales used by Antebi-Gruszka and Scheer (41) and Scheer and Poteat (44) can be associated with the principle of *trustworthiness and transparency*, such as “I can trust staff.” (47). The previously mentioned category “emotional trust and safety” from the thematic analysis by Hall and DeLaney (42) includes not only the SAMHSA (15) principle of *safety* but also *trustworthiness and transparency*. The latter is reflected in themes such as predictability, dependability, and reliability, as well as a nonjudgmental attitude from therapists. In addition, Lacombe-Duncan et al. (43) found that it is essential to enhance trust and comfort through an affirmative, non-stigmatizing, respectful, and nonjudgmental approach, thus allowing clients the opportunity to discuss potentially stigmatizing topics.

The questions from the subscale “Connection” of the TIP Scales used by Antebi-Gruszka and Scheer (41) and Scheer and Poteat (44) can be associated with the SAMHSA (15) principle of *peer support* [e.g., “In this program, I have the opportunity to connect with others.” (47)]. This principle was not specifically addressed in the thematic analysis by Hall and DeLaney (42), and similarly, Lacombe-Duncan et al. (43) do not explicitly mention *peer support* or corresponding resources. However, the latter note that it can be beneficial to discuss difficult topics with friends or HCPs at eye level, which could be categorized as *peer support* in a broader sense.

Hall and DeLaney (42) emphasize the category “Choice and Collaboration,” which aligns well with the TIC principle *collaboration and mutuality* according to SAMHSA (15). Topics include therapeutic self-disclosure as a means of altering power imbalances and the importance of reducing power disparities at the community level (e.g., due to differences in gender identity or ethnicity) (42). Along similar lines, Lacombe-Duncan et al. (43) elaborate in their qualitative analysis that it is important to recognize the power and privilege of HCPs and actively deconstruct these dynamics. The subscale “Information” of the TIP Scales used by Antebi-Gruszka and Scheer (41) and Scheer and Poteat (44) cannot be clearly assigned to a specific principle (e.g., “I have the opportunity to learn how abuse and other difficulties affect responses in the body.” (47)) and may best align with aspects of this TIC principle, as the provision of information can reduce power inequalities and facilitates collaboration on equal terms.

Hall and DeLaney (42) found that participants perceive it as “empowerment” when counselors advocate for them and facilitate access to medical interventions and when they learn skills for personal development during therapy to better manage their daily lives. In Lacombe-Duncan et al. (43), aspects of this principle are evident in the reports from trans women, who positively describe how HCPs allow them to decide which tests and prescriptions they need, rather than assuming or deciding what is best for them. Furthermore, some items from the subscale “Agency” of the TIP Scales used by Antebi-Gruszka and Scheer (41) and Scheer and Poteat (44), such as “Staff respect the choices that I make.” (47) can also be associated with the SAMHSA (15) principle of *empowerment, voice, and choice*. Additionally, the subscale “Strengths” also aligns with this principle, featuring items such as “Staff respect the strengths I have gained through my life experiences.” (47).

The subscale “Cultural Responsiveness and Inclusivity” within the TIP Scales used by Antebi-Gruszka and Scheer (41), can be aligned with the SAMHSA (15) principle of *cultural, historical, and gender issues* (e. g. “Staff understand the challenges faced by people who are immigrants.” (47)). Hall and DeLaney (42) identify the category “cultural and gender issues,” emphasizing that HCPs should value the subjective experiences of clients and refrain from imposing their own perceptions onto them. Furthermore, they stress that ongoing education for HCPs (and the community) regarding TGD is essential for providing quality care and finding a good way of dealing with this topic within the community. Lacombe-Duncan et al. (43) highlight that HCPs should account for the potential discrimination and violence individuals have experienced within health-care settings. Additionally, they state that the unique characteristics of this group should be contextualized and understood as potential products of gender-based trauma.

### 3.3 Study results related to Trauma-Informed Care

The analyzed studies present mixed results regarding the application for TIC in the context of healthcare for TGD individuals. Antebi-Gruszka and Scheer (41) found that enhancement of the component “Agency” was associated with reduced depression, PTSD, shame, and loneliness. However, they also found “Connection” to be positively associated with sexual risk and the component “Cultural Responsiveness and Inclusivity” to depression, PTSD, and chronic physical health problems. The found no significant links for “Information”, “Strengths”, or “Minority Stress” with the analyzed outcomes. Scheer and Poteat (44) found TIC to be significantly related to greater empowerment, emotion regulation, and lower social withdrawal, while not finding the hypothesized indirect associations between TIC and mental or physical health through the examined mechanisms. Only the indirect association of TIC to mental health through lower social withdrawal was significant. Furthermore, Hall and DeLaney (42) found in their analysis that the results of the qualitative survey could be fitted into the framework of TIC. They applied five of the SAMHSA (15) elements of TIC to the results and came to the conclusion that TIC provides a framework for the needs of TGD people within mental health and community support and can therefore be a way of better meeting these needs. Lacombe-Duncan et al. (43) also identified aspects of TIC within their qualitative data. While different elements of TIC were not explicitly differentiated according to SAMHSA (15), they determined TIC to be the most prominent facilitator for access to HIV care for trans women that emerged from the thematic analysis.

## 4 Discussion

The four reviewed studies showed differing results regarding the application of TIC in the care for TGD people. Antebi-Gruszka and Scheer (41) found both positive and negative associations between TIC and health outcomes, while Scheer and Poteat (44) reported only positive associations. Hall and DeLaney (42) and Lacombe-Duncan et al. (43) identified TIC elements in their qualitative data, highlighting TIC as a facilitator for accessing HIV care and addressing the needs of transgender and gender-diverse individuals. The findings of Hall and DeLaney (42) appear particularly relevant as the responses to the open-ended (non-TIC-specific) questions about health-care needs fit so well into the TIC framework that it was applied in their analysis. This alignment suggests that the core principles of TIC naturally address the needs and concerns of TGD individuals within the health-care system. TGD individuals experience a distinct set of health-care needs stemming from their unique vulnerabilities and experiences, such as discrimination. Key needs include safe and affirming health-care environments where their gender identity is recognized and respected (9, 10). The fact that participants’ descriptions of their needs in the findings of Hall and DeLaney (42) organically matched the TIC categories highlights the framework’s inherent suitability in meeting these needs. These findings hint a potential for TIC to be a beneficial approach in various health-care contexts and specifically in the care for TGD individuals.

The current analysis suggests a potential of TIC in healthcare for TGD people to meet the needs of this understudied group. Given the significant prevalence of traumatic experiences and discrimination within this population (13, 14), HCPs should understand and address the impacts of trauma and minority stress to improve health outcomes (30–33). Although research on TIC in the context of TGD care remains limited, the existing studies hint towards promising results. Given these considerations, it may be worth investigating what implications TIC could have for other approaches or paradigms in the field. Importantly, TIC is not a stand-alone intervention but a flexible framework that can be integrated with a wide range of therapeutic modalities and care models (15). This adaptability makes it especially suitable for combination with other approaches, potentially enhancing their responsiveness to the lived experiences of TGD individuals. Thus, TIC could, for example, be implemented in areas where authors have already advocated for the adoption of this framework, such as reproductive health-care settings (30), pediatrics (31), imaging care (32), and mental healthcare (33). Moreover, to some degree TIC aligns conceptually with approaches such as LGBTQ-affirmative cognitive-behavioral therapy (48, 49), minority stress-informed approaches (50), and intersectionality-based framework (51), and could therefore relatively easily be integrated into these models and potentially enhance existing care paradigms. Exploring such integrative applications could support the development of more holistic, context-sensitive, and suitable practices in TGD healthcare. However, the findings of this review suggest that, despite some recommendations in the literature, TIC has so far been only sparsely researched and its implementation in TGD healthcare remains limited. As mentioned before, in current guidelines TIC is not explicitly mentioned although the principles seem to have informed some of their recommendations (1, 34–36). The preliminary evidence (42, 44) and the theoretical benefits (15, 24) discussed in this review suggest that further exploration and development of TIC in TGD care could be beneficial. Should more high-quality evidence become available, the inclusion of TIC in health-care guidelines could be considered as one possible step toward supporting its implementation. Further rigorous studies specifically examining the effectiveness of TIC in TGD care are needed to inform such considerations. Strengthening the evidence base will be important for determining whether and how TIC may contribute meaningfully to care for this population.

When looking at the SAMHSA (15) principles separately it appears that *safety* is critical in the TIC framework. Antebi-Gruszka and Scheer (41) found that higher levels of the respective subscale were linked to lower levels of depression, PTSD, shame, and loneliness, suggesting that enhancing *emotional safety* may positively impact mental health outcomes. However, the interpretability of these results is limited, as the subscale consists of items that can be assigned to several SAMHSA (15) principles. It could be argued that *emotional safety*, *trustworthiness and transparency*, and *empowerment, voice, and choice* collectively contribute to the observed positive association with mental health outcomes. Additionally, it should be emphasized that, despite *physical safety* being a critical need for TGD individuals within the health-care system (9, 10), the TIP Scales do not include items addressing this aspect, and Lacombe-Duncan et al. (43) also do not explicitly mention it. Highlighting this need, Hall and DeLaney’s (42) findings distinguish between *emotional* and *physical safety*, indicating that TGD individuals regard both facets of the construct as crucial in their care. Furthermore, the principle *trustworthiness and transparency and empowerment, voice, and choice* were identified across all analyzed studies. *Collaboration and mutuality* was particularly highlighted as an important factor in the qualitative studies (42, 43). This factor, however, was not explicitly assessed in the quantitative studies, although it can be inferred from some items (47). Even though the results on this are thus incomplete, the qualitative studies indicate that this factor is regarded as important by both TGD individuals and HCPs, as both studies emphasize the need to deconstruct power imbalances in the relationship between clients and HCPs. Given that TGD individuals represent a marginalized group that often experiences powerlessness in society (10), it is evident that this aspect is emphasized in the studies, as it addresses a crucial need for TGD individuals in care settings. The SAMHSA (15) principle of *cultural, historical, and gender issues* was found in all analyzed studies apart from Scheer and Poteat (44) and *peer support* was only present in the two quantitative studies (41, 44). Antebi-Gruszka and Scheer (41) found negative results for the subscale “Connection” which was associated positively with sexual risk and “Cultural Responsiveness and Inclusivity” with depression, PTSD, and chronic physical health problems. These results underscore the importance of nuanced and targeted approaches in implementing TIC to maximize its benefits and minimize potential adverse effects. They can contribute to an enhanced understanding of TIC components and provide critical information for HCPs in the care of TGD individuals. Examples include focusing on STI-risk behavior (due to the positive association between “Connection” and sexual risk) or ensuring that when emphasizing the TIC component *cultural, historical, and gender issues*, it does not reinforce culturally induced stigmatization (due to the positive association with negative health outcomes) (52). Ultimately, because of the limited and mixed results available the different components should be further examined.

It is important to highlight that the studies analyzed do not provide data on the effectiveness or efficacy of specific trauma-informed interventions, as studies that would allow for such conclusions – such as randomized controlled trials – are currently absent in the literature. Consequently, no definitive conclusions can be drawn about the effectiveness or efficacy of TIC approaches in this context. The reported perceptions and outcomes, which are based on common study designs in health services research, may offer valuable practice-oriented insights, but they do not establish the direct effectiveness of TIC as would randomized clinical trials. This clear gap underscores the need for future research to systematically assess the impact of TIC strategies across diverse health-care settings. Furthermore, in the analysis of the studies, it becomes evident that the assessment of TIC was highly heterogeneous, which significantly complicates comparability and interpretability of the studies. For example, Scheer and Poteat (44) did not consider aspects of *cultural, historical, and gender issues*, while Hall and DeLaney (42) and Lacombe-Duncan et al. (43) did not include *peer support* in their analyses. At the same time, Scheer and Poteat (44) and Antebi-Gruszka and Scheer (41) incorporated minority stress TIC in their analysis, adding to the classical SAMHSA (15) principles. Furthermore, Antebi-Gruszka and Scheer (41) and Hall and DeLaney (42) placed an increased focus on the individual components of TIC, whereas Scheer and Poteat (44) included TIC as a whole in their analysis, and Lacombe-Duncan et al. (43) did not explicitly highlight the SAMHSA (15) principles. These differences could account for the varying results. Furthermore, while the varying research questions and methodologies across the analyzed studies limit the comparability, it demonstrates that TIC can be assessed using both quantitative and qualitative methods and highlights the versatility of the TIC framework in capturing a broad spectrum of data. The discussed findings from the analyzed studies underscore several critical areas for future research on TIC, highlighting the need for more nuanced and comprehensive studies to advance the understanding of the effects of TIC. To overcome inconsistencies in TIC definitions and applications, future research should work towards establishing a unified definition and framework for TIC, building on SAMHSA (15). Furthermore, future studies should develop and utilize standardized outcome measures to assess all facets of TIC. This would facilitate more robust comparisons across different studies and contribute to a better understanding of TIC’s overall effects.

In addition, the studies examined TIC in varied contexts, including services related to the survival of intimate partner violence such as therapy or legal counselling (41, 44), mental health and community support (42), and access to HIV care (43). This diversity in context examined in the analyzed studies underscores the adaptability and relevance of the TIC framework across different settings and populations. The findings of Lacombe-Duncan et al. (43) are especially noteworthy because their study identified TIC elements as significant facilitators for accessing HIV care among trans women, suggesting that trauma-informed approaches can be applied in TGD care beyond mental health and psychosocial outcomes. This adaptability underscores the potential of TIC to be integrated into different service settings, enhancing support and care for diverse populations. Notably, Lacombe-Duncan et al. (43) also included HCPs in their survey, which can give a more complete and nuanced view of TIC, as it also considers the perspective of the people providing care. Moreover, the positive results of the analysis involving HCPs support the implementation of TIC, as it appears to be well-received and applicable from the HCPs’ perspective. This is further supported by Ferguson and Maccio (37), who analyzed 19 non-profit organizations in the USA serving TGD individuals and found that TIC was already frequently used within this context. Incorporating HCPs’ perspectives in future research is essential, as it can yield valuable insights into the practical implementation of TIC and help identify potential barriers and facilitators that may influence its effects. In future research TIC should be studied across diverse populations and service contexts to enhance the understanding of TIC. Furthermore, the studies included a range of populations, with some focusing specifically on TGD individuals (42) and others encompassing broader LGBTQ populations (e.g., Scheer and Poteat (44)). This variation enriches the overall understanding of the experiences within these communities. However, while the experiences and needs of TGD individuals can resemble those of the wider LGBTQ community, there may be specific needs in the care of TGD individuals that should be considered (53). Therefore, future research should aim to clearly delineate these groups, ensuring that findings are accurately attributed and that interventions are appropriately designed to address the distinct needs of TGD populations.

In their definition of TIC, SAMHSA (15) highlight another point that has not been considered in the analyzed studies or in much of existing research in the context of TIC. This is the collaboration between different sectors of the health-care system. According to SAMHSA (15), cross-sector collaboration relies on a shared understanding of trauma and the principles of a trauma-informed approach to ensure that different sectors work together effectively to meet the complex needs of individuals with significant trauma histories. TGD individuals and especially individuals with histories of trauma or prolonged minority stress often have multifaceted needs that span various service sectors, including mental health, endocrinology, urology, surgery, and legal services (1). When these needs are addressed in isolation, there is a risk that trauma-sensitive progress made in one sector can be undermined by the insensitivity or lack of understanding in another sector (15). In building on the insights from SAMHSA (15) about the importance of cross-sector collaboration, future research should explore strategies for enhancing TIC implementation across different service sectors, developing models for cross-sector collaboration that ensure continuity of care.

### 4.1 Limitations

There are several limitations to this scoping review that should be considered when interpreting the results. One is the methodological diversity described for assessing TIC as well as regarding the outcomes among the studies. This complicates the synthesis of results, limits the comparability of the studies, and makes the process prone to errors. Likewise, the lack of a standardized and official definition for TIC limits the comparability. Another limitation lies in the variability of study populations and contexts described in the previous. The range of contexts, from intimate partner violence to HIV care, illustrates the adaptability of TIC but also means that findings from individual studies might not be universally applicable across different domains of care. Additionally, the studies included populations with varying degrees of representation of TGD individuals versus broader LGBTQ groups. These differences in populations mean that findings from one study might not be generalizable to the populations studied in others. Additionally, all four studies included analyzed an adult sample (18 years and older). Therefore, no conclusions can be drawn regarding the application of TIC for TGD children and adolescents. A further limitation is the predominantly North American context of the included studies, with mostly White participants, which may limit the generalizability of the findings to other cultural or geographical settings. Additionally, information on socioeconomic background was only available for two studies and indicated that the sample was mainly middle-class, further restricting transferability to more diverse populations. These limitations impair the generalizability of the results of this scoping review.

Furthermore, it is important to recognize that this review did not involve a formal assessment of study quality as this is not in the sense of a scoping review in order to avoid the exclusion of relevant studies and to obtain a comprehensive overview of the research area (39). However, this means that the possible qualitative limitations (e. g. reliance on self-report measurements, not validated questionnaires, sample size, sampling methods) in the studies analyzed could not be accounted for and represent a substantial limitation for the interpretability of the results. In addition, the study screening and selection process was only done by one person, which makes it more prone to errors.

## 5 Conclusion

This scoping review highlights both the potential and the complexities of the TIC framework in addressing the needs of TGD people. The reviewed studies indicate that TIC may have the potential to meet the needs of TGD people and may offer benefits for their care but also indicate that its impact might not be uniformly positive. Methodological differences, varying definitions of TIC, and various outcomes as well as the available literature and research limit the extent to which study results can be compared and generalized. This review underscores the need for future research to adopt standardized measures, examine different populations, conduct intervention studies, and explore TIC’s application across different service sectors. In conclusion, the findings from the reviewed studies point towards a potential of TIC in the care for TGD individuals, while also raising numerous questions and directions for future research in this field.
